# Probiotic *Enterococcus faecium* (M74) as an alternative to antibiotics for controlling necrotic enteritis in broiler chickens

**DOI:** 10.1038/s41598-026-42376-4

**Published:** 2026-03-23

**Authors:** Ahmed E. A. Mostafa, Rana Ramadan, Ahmed Sittien

**Affiliations:** 1 Department of Preclinical Veterinary Medical Sciences (Pharmacology), Faculty of Veterinary Medicine, Delta University for Science and Technology, Gamasa City, Dakahlia Governorate Egypt; 2 Department of Basic Veterinary Science, Faculty of Veterinary Medicine, Delta University for Science and Technology, Mansoura, Egypt; 3 Department of Preclinical Veterinary Medical Sciences, Faculty of Veterinary Medicine, Delta University for Science and Technology, Gamasa City, Dakahlia Governorate Egypt

**Keywords:** Broilers, Clostridium perfringens, *Enterococcus faecium* (M74), Necrotic enteritis, Probiotic, Antibiotic alternative, Microbiology, Zoology

## Abstract

Necrotic enteritis caused by *Clostridium perfringens* remains a major challenge in broiler production, particularly under restrictions on antibiotic use. This study evaluated the prophylactic and therapeutic effects of *Enterococcus faecium* (M74) as a probiotic alternative to antibiotics in broiler chickens experimentally challenged with *C. perfringens*. Birds were allocated into negative control, positive control, prophylactic, and therapeutic groups, and growth performance, bacterial counts, hematological and biochemical indices, immune responses, intestinal morphometry, and histopathology were assessed. Supplementation with *E. faecium* (M74) markedly reduced intestinal bacterial load, with *C. perfringens* and total coliform counts decreased by approximately 88% and 84%, respectively, in the prophylactic group compared with the infected control. Final body weight and body weight gain were improved by approximately 30–31%, while feed conversion ratio was improved by about 25%. Villus height increased by approximately 43%, accompanied by a marked reduction in histopathological lesion scores (up to 82% decrease in degeneration score). Renal and hepatic function markers were significantly ameliorated, and immune parameters were markedly enhanced in treated birds. The prophylactic regimen consistently showed superior efficacy compared with therapeutic application. Although the study was limited to a single probiotic strain and experimental conditions, these findings indicate that *E. faecium* (M74) is a promising probiotic candidate for mitigating necrotic enteritis in broilers and may represent a viable alternative or complementary strategy to antibiotics.

## Introduction

Enteric diseases represent a major challenge for the poultry industry because of their association with economic losses, impaired growth performance, increased mortality, and compromised animal welfare, in addition to the potential contamination of poultry products intended for human consumption. Among these diseases, necrotic enteritis (NE) is considered one of the most economically important enteric disorders in broiler production worldwide. The disease is primarily caused by *Clostridium perfringens*, a Gram-positive, spore-forming anaerobic bacterium that produces several toxins, including the avian-specific necrotic enteritis beta toxin (NetB), which plays a key role in intestinal tissue damage and disease pathogenesis^1^.

For several decades, antibiotics have been widely used in poultry production to improve growth performance, enhance feed efficiency, and control enteric bacterial infections. Field observations have shown that even low dietary levels of antibiotics such as lincomycin can markedly reduce the incidence of NE^2^. However, the long-term and extensive use of antibiotics has contributed to the emergence and spread of antibiotic-resistant bacteria, raising serious concerns among scientists, regulatory authorities, and consumers^3^. Antimicrobial resistance (AMR) is now recognized as a major global public health threat, and increasing restrictions on the use of antibiotics in animal production have intensified the search for effective and sustainable alternatives^4^.

In this context, probiotics have gained considerable attention as promising non-antibiotic strategies for maintaining gut health and improving disease resistance in poultry. Probiotics are defined by the Food and Agriculture Organization (FAO) and the World Health Organization (WHO) as “live microorganisms which, when administered in adequate amounts, confer a health benefit on the host”^5^. An effective probiotic strain should be able to survive gastric acidity and bile salts, colonize the gastrointestinal tract, exert antimicrobial or competitive exclusion effects against pathogens, and remain viable during processing, storage, and field application, while also being economically feasible for large-scale use.

Despite their potential benefits, the use of Enterococcus-based probiotics requires careful safety consideration. Certain Enterococcus strains are known to harbor virulence factors and antibiotic resistance genes, and there is a theoretical risk of horizontal gene transfer to other members of the intestinal microbiota. Such gene transfer could contribute to the dissemination of undesirable traits within the gut ecosystem, which raises legitimate safety concerns. Therefore, the selection of Enterococcus strains for probiotic use should be based on rigorous safety evaluation, and their efficacy and safety should be confirmed under in vivo conditions.

*Enterococcus faecium* (M74), a lactic acid bacterium and a natural inhabitant of the gastrointestinal tract, has been proposed as a promising probiotic candidate in animal production. Previous studies have reported that dietary inclusion of *E. faecium* (M74) can reduce fecal shedding of *Clostridium perfringens* and increase populations of beneficial bacteria such as Bifidobacterium and Lactobacillus spp^6^. These findings suggest a potential role for this strain in improving gut microbial balance and enhancing resistance to enteric infections.

However, most available studies have focused either on general performance parameters or on in vitro antimicrobial activity, with limited in vivo evidence addressing its protective efficacy against NE under experimental challenge conditions. More importantly, there is a lack of comprehensive studies directly comparing the prophylactic and therapeutic application of *E. faecium* (M74) against *C. perfringens*-induced necrotic enteritis in broiler chickens, while simultaneously evaluating growth performance, immune responses, intestinal morphology, histopathological alterations, and bacterial load. This represents a clear gap in the current literature, particularly in the context of identifying safe and effective alternatives to antibiotics for NE control.

Therefore, the present study was designed to evaluate the prophylactic and therapeutic efficacy of *Enterococcus faecium* (M74) in broiler chickens experimentally challenged with *Clostridium perfringens* and to provide an integrated assessment of its effects on growth performance, hematological and biochemical parameters, immune responses, intestinal morphology, histopathology, and bacterial re-isolation.

## Materials and methods

### ARRIVE guidelines and ethics approval

This study was conducted in accordance with the ARRIVE guidelines (Animal Research: Reporting of In Vivo Experiments). All experimental procedures involving animals complied with institutional and international standards for animal care and use.

The experimental protocol was reviewed and approved by the Institutional Animal Care and Use Committee (IACUC), Faculty of pharmacy, Delta University for Science and Technology, Egypt (Approval No.: FPDU36/2026).

All experimental work was performed at the Laboratory Animal Facility, Faculty of Veterinary Medicine, Zagazig University, Egypt, under an institutional cooperation agreement. Animal handling, anesthesia, and euthanasia procedures adhered to the ethical standards of both institutions.

Every effort was made to minimize animal suffering, reduce stress, and use the minimum number of animals required to achieve statistical validity.

### Materials

#### Enterococcus faecium M74

The product *Enterococcus faecium* M74 is provisionally authorized under Council Directive 70/524/EEC as a feed additive in the category “Micro-organism” for use in calves and piglets until 6 January 2004 (first authorization: Commission Regulation (EC) No. 2690/1999). The Scientific Committee on Animal Nutrition (SCAN) issued a favorable opinion on the safety of “Lactiferm” for these animal categories. Medipharm AB (Sweden) sought authorization for its microorganism product *E. faecium* M74 as a feed additive intended for chickens for fattening under the same category.

*Enterococcus faecium* M74 (NCIMB 11181) is a microbial probiotic belonging to the “Micro-organism” product category and is specifically intended for use in chickens for fattening. The product was developed and submitted for authorization by Medipharm AB (Sweden). In the present study, dehydrated *E. faecium* M74 was administered via drinking water at a dose equivalent to 1.5 × 10⁹ CFU kg⁻¹, following previously reported recommendations^8^.

##### Inoculum preparation and challenge

On day 19, all infected groups were orally inoculated with 1 mL of *Clostridium perfringens* type A field strain (1.9 × 10⁹ organisms/mL), obtained from the Anaerobes Unit, Animal Health Research Institute, Dokki, Giza, Egypt^9^. The reference strain (*C. perfringens* type A, ATCC™ 13124™) was cultured in meat broth and incubated for 24–48 h at 37 °C under anaerobic conditions using a GasPak system. The bacterial cells were then resuspended in phosphate-buffered saline (PBS).

##### Justification of Probiotic Dose

The selected dose (1 g/L drinking water; equivalent to 1.5 × 10⁹ CFU/kg) was based on manufacturer recommendations and supported by previous peer-reviewed studies demonstrating optimal intestinal colonization, pathogen inhibition, and immunostimulatory activity without adverse effects. This concentration has been reported to provide sufficient viable bacterial counts to withstand dilution in drinking water while ensuring effective delivery to the gastrointestinal tract.

##### Confirmation of Probiotic Viability in Drinking Water

Probiotic stability during water administration, freshly prepared drinking water supplemented with *Enterococcus faecium* M74 (1 g/L) was prepared daily. Immediately after preparation (0 h), and at 6 and 12 h post-dilution, 1 mL water samples were aseptically collected from drinkers and subjected to microbiological analysis. Samples were serially diluted (10-fold) in sterile phosphate-buffered saline and plated in duplicate onto de Man, Rogosa, and Sharpe (MRS) agar. Plates were incubated aerobically at 37 °C for 24 h, after which colonies were counted and expressed as CFU/mL. Viable counts remained above 10⁸ CFU/mL throughout the monitoring period, indicating that the probiotic maintained adequate viability under the experimental housing conditions. Drinking water was replaced every 12 h, and drinkers were thoroughly rinsed and air-dried before refilling to prevent biofilm formation and unintended bacterial proliferation.

#### Experimental birds

A total of 120 one-day-old commercial Hubbard broiler chicks (38–42 g) were obtained from Al-Kahira Poultry Company (10th of Ramadan City, Egypt). Chicks were reared under standard hygienic and environmental conditions, with feed and water provided *ad libitum*. All birds were vaccinated against Newcastle disease on days 7 and 18 using Hitchner B1 and LaSota live virus vaccines (Intervet Boxmeer, Netherlands; 10⁶ EID₅₀/dose, reconstituted in 30 mL physiological saline per 1000 doses, applied as eye drops). Vaccination against Gumboro disease was carried out on day 15 using the Holland Gumboro vaccine (Rhone-Mérieux, France; reconstituted in 50 mL saline per 1000 birds). The experiment was conducted in the Laboratory Animal Facility, Faculty of Veterinary Medicine, Zagazig University, Egypt, under temperature conditions of 25 ± 2 °C. All managemental practices were followed to keep the birds free from stress. The study was approved by the Institutional Animal Care and Ethics Committee of Zagazig University, Egypt.

#### Experimental design

The experiment included four groups (30 birds each):

1. **Negative control**: Non-infected birds fed a basal diet only.
2. **Positive control**: Birds fed a basal diet and orally infected with 2 mL of *C. perfringens* broth culture (1.9 × 10⁹ organisms/mL) on day 19^9^.
3. **Prophylactic** ***E. faecium*** **M74 group**: Birds received dehydrated *E. faecium* M74 (1 g/L in drinking water, 1.5 × 10⁹ CFU kg⁻¹) from day 1 to the end of the experiment and were infected with *C. perfringens* on day 19^8^.
4. **Therapeutic** ***E. faecium*** **M74 group**: Birds were infected with *C. perfringens* on day 19 and subsequently treated with *E. faecium* M74 (1 g/L in drinking water, 1.5 × 10⁹ CFU kg⁻¹) for five consecutive days following the appearance of clinical signs.

#### Sampling

##### Anesthesia and euthanasia procedures

Prior to sampling and necropsy, broiler chickens were anesthetized using a ketamine–xylazine combination. Ketamine was administered at a dose of 35 mg/kg body weight and xylazine at 5 mg/kg body weight via intramuscular injection into the thigh muscles. This protocol ensured adequate analgesia and loss of reflex response during handling and tissue collection. The birds were 35 days old at the time of anesthesia, with an average body weight of 1.8–2.2 kg.

At the end of the experiment, euthanasia was humanely performed in accordance with AVMA and institutional animal welfare guidelines. Birds were euthanized by cervical dislocation following deep anesthesia to ensure complete loss of consciousness prior to death. This method is recognized as an approved and humane procedure for poultry research studies.

##### Blood samples

Blood samples were collected from five birds per group on days 2, 9, and 16 post-infection (prophylactic group) and on days 2, 9, and 16 post-treatment (therapeutic group). Each sample was divided into two portions:

- One collected on dipotassium EDTA (1 mg/mL blood) for hematological analysis.
- The second collected in plain centrifuge tubes to obtain serum for biochemical and immunological assays^10^.

##### Tissue samples

Segments of small intestine were collected post-sacrifice for histopathological examination.

### Methods

#### Hematological analysis

Total erythrocyte and leukocyte counts and hemoglobin concentration (Hb) were determined using an Auto Hematology Analyzer (Rayto 7200, MEDINICS R).

#### Biochemical analysis (liver function)

Serum levels of AST, ALT, ALP, total protein, albumin, and globulin were measured using a semi-automated clinical chemistry analyzer (Chem 7, Erba R) according to^11^.

#### Kidney function tests

Serum concentrations of urea, creatinine, uric acid, and potassium were determined using the same analyzer and following^12^.

#### Immunological parameters

Serum immunoglobulins (IgM, IgA), cytokines (IL-2, IL-4, TNF-α), and T-cell markers (CD4, CD8) were quantified using ELISA kits (ChroMate 4300, Microplate Reader, Biotek India) following manufacturer instructions. Absorbance at 450 nm (A₄₅₀) was recorded, and antibody concentrations were calculated from calibration curves.

#### Growth performance

Growth parameters including body weight (BW), body weight gain (BWG), feed intake (FI), and feed conversion ratio (FCR) were monitored weekly.

- **BWG** = Final BW − Initial BW.
- **FCR** = Feed intake (g)/Weight gain (g).

#### Histopathological examination

Intestinal tissues were fixed in 10% neutral buffered formalin for 12 h, dehydrated through graded ethanol, cleared in xylene (1:1), and embedded in paraffin wax (melting point 56 °C). Section (5 μm) were cut using a microtome (Leica RM2255, Germany), stained with hematoxylin and eosin (H&E), and examined microscopically (Olympus CX41, Tokyo, Japan). Procedures followed^14^.

#### Re-isolation of *C. perfringens*

Intestinal samples (*n* = 10 per group) were collected at 35 days of age. Serial 10-fold dilutions were prepared, and 0.1 mL aliquots were plated on blood agar (5% sheep blood + 100 mg/L neomycin sulfate). Plates were incubated anaerobically at 37 °C for 16–24 h. α- and β-hemolytic colonies were counted and identified by Gram staining. Counts were expressed as log₁₀ CFU/g intestinal content^15^.

#### Statistical analysis

Data were analyzed using SPSS (version 25.0, IBM Corp., Chicago, IL, USA) and GraphPad Prism (version 9.4). One-way ANOVA was applied, and results were expressed as mean ± standard error (SE). Statistical significance was set at *p* ≤ 0.05.

## Results

### Hematological parameters

Supplementation with *Enterococcus faecium* M74 (15 × 10⁹ CFU/kg) markedly improved the hematological profile of broilers challenged with *Clostridium perfringens* type A (1.9 × 10⁹ CFU/mL). As shown in Table 1, both prophylactic and therapeutic groups exhibited significant increases in total white blood cell (WBC) count, red blood cell (RBC) count, hemoglobin concentration (Hb), and packed cell volume (PCV) compared with the positive control group (*p* ≤ 0.05). For example, WBC increased from 14.2 ± 0.5 × 10⁹/L in the positive control to 17.5 ± 0.3 × 10⁹/L in the prophylactic group. Conversely, mean corpuscular volume (MCV), mean corpuscular hemoglobin (MCH), and mean corpuscular hemoglobin concentration (MCHC) remained within normal physiological ranges, indicating overall improvement in blood physiology and oxygen-carrying capacity. These results suggest that early administration of *E. faecium* M74 supports hematological homeostasis and enhances immune readiness during *C. perfringens* infection.

**Table 1:** Effect of *Enterococcus faecium* M74 on hematological parameters in broilers after *C. perfringens* type A infection (19th day).

Parameter	Negative Control	Positive Control	Prophylactic	Therapeutic
WBC (×10⁹/L)	14.2 ± 0.5 ^b^	15.8 ± 0.2 ^ab^	17.5 ± 0.3^a^	15.5 ± 0.3^ab^
RBC (×10¹²/L)	2.5 ± 0.04^bc^	2.9 ± 0.03 ^a^	2 ± 0.02 ^c^	3.1 ± 0.33^a^
Hb (g/L)	7.5 ± 0.1 ^d^	8.4 ± 0.01 ^a^	6.4 ± 0.01 ^e^	9.0 ± 0.13 ^a^
PCV (%)	24.2 ± 4 ^b^	26 ± 3 ^a^	21.5 ± 2 ^d^	25.5 ± 3 ^ab^
MCV (fL)	150 ± 4 ^a^	145 ± 1 ^b^	145 ± 4 ^b^	95 ± 3 ^c^
MCH (pg)	47 ± 3 ^b^	45 ± 3 ^c^	50 ± 2 ^a^	33 ± 2 ^d^
MCHC (g/L)	40 ± 1.5 ^a^	28 ± 2.1 ^c^	45 ± 1.9 ^a^	27 ± 1 ^c^

Values are mean ± SD. Different superscript letters (a-d) indicate significant differences among groups (p ≤ 0.05, one-way ANOVA followed by Duncan’s multiple range test).

### Liver Function Parameters

Supplementation with *Enterococcus faecium* M74 (15 × 10⁹ CFU/kg) significantly alleviated hepatic damage caused by *Clostridium perfringens* type A (1.9 × 10⁹ CFU/mL). As shown in Table 2, both prophylactic and therapeutic groups exhibited significantly lower serum AST, ALT, and ALP levels (*p* ≤ 0.05) compared with the positive control, approaching the values of the negative control. For example, AST decreased from 82.4 ± 7.2 U/L in the positive control to 51.1 ± 1.3 U/L in the prophylactic group.

Additionally, total protein, albumin, and globulin levels were significantly restored in the treated birds. Total protein increased from 3.36 ± 0.2 g/dL in the negative control to 3.7 ± 0.05 g/dL in the prophylactic group, while albumin and globulin showed similar restoration patterns. These findings reflect improved hepatic function and reduced hepatocellular leakage, indicating that *E. faecium* M74 effectively supports liver health during C. perfringens infection.

**Table 2:** Effect of *Enterococcus faecium* M74 on liver function parameters in broilers after *C. perfringens* type A infection (19th day).

Parameter	Negative Control	Positive Control	Prophylactic	Therapeutic
AST (U/L)	33.8 ± 1.5 ^c^	82.4 ± 7.2 ^a^	51.1 ± 1.3 ^b^	55.8 ± 2.4 ^b^
ALT (U/L)	23.2 ± 2.5 ^c^	58.4 ± 3.6 ^a^	35.6 ± 1.4 ^b^	38.3 ± 1.8 ^b^
ALP (U/L)	48.6 ± 1.2 ^c^	137.4 ± 6.9 ^a^	82.4 ± 2.4^b^	88.6 ± 1.9 ^b^
Total protein (g/dL)	3.36 ± 0.2 ^c^	6.8 ± 0.01 ^a^	3.7 ± 0.05 ^b^	3.5 ± 0.4 ^b^
Albumin (g/dL)	3.20 ± 0.21 ^b^	4.9 ± 0.1 ^a^	3.2 ± 0.05 ^b^	2.9 ± 0.6 ^b^
Globulin (g/dL)	0.88 ± 0.16 ^b^	0.8 ± 0.01^b^	0.92 ± 0.06 ^a^	0.91 ± 0.06 ^a^

Values are mean ± SE (n = 5). Different superscripts (a, b, c) indicate significant differences among groups (p ≤ 0.05, one-way ANOVA followed by Duncan’s multiple range test).

### Kidney function parameters

Supplementation with *Enterococcus faecium* M74 (15 × 10⁹ CFU/kg) significantly protected renal function in broilers challenged with *Clostridium perfringens* type A (1.9 × 10⁹ CFU/mL). As shown in Table 3, the positive control group exhibited elevated levels of serum urea, creatinine, uric acid, and potassium, indicating impaired kidney function. Both prophylactic and therapeutic groups demonstrated significant reductions in these parameters (*p* ≤ 0.05), approaching values of the negative control.

For example, urea decreased from 46.7 ± 3.41 mmol/L in the positive control to 29.6 ± 2.32 mmol/L in the prophylactic group, and creatinine decreased from 1.34 ± 0.21 mg/dL to 0.85 ± 0.13 mg/dL. These results suggest that *E. faecium* M74 exerts a protective effect on renal physiology during C. perfringens infection.

**Table 3:** Effect of *Enterococcus faecium* M74 on kidney function parameters in broilers after *C. perfringens* type A infection (19th day).

Parameter	Negative Control	Positive Control	Prophylactic	Therapeutic
Urea (mmol/L)	28.8 ± 1.20 ^b^	46.7 ± 3.41 ^a^	29.6 ± 2.32 ^b^	29.8 ± 1.28 ^b^
Creatinine (mg/dL)	0.77 ± 0.12 ^c^	1.34 ± 0.21 ^a^	0.85 ± 0.13 ^b^	0.91 ± 0.16 ^b^
Uric acid (mg/dL)	2.52 ± 0.04 ^b^	5.61 ± 0.06 ^a^	2.85 ± 0.60 ^b^	2.97 ± 0.07 ^b^
Potassium (mg/dL)	135.5 ± 1.0 ^b^	150.3 ± 1.8 ^a^	138.0 ± 1.8 ^b^	138.9 ± 1.7 ^b^

Values are mean ± SE (n = 5). Different superscripts (a–c) indicate significant differences among groups (p ≤ 0.05, one-way ANOVA followed by Duncan’s multiple range test).

### Immunological parameters

Supplementation with *Enterococcus faecium* M74 (15 × 10⁹ CFU/kg) significantly enhanced both humoral and cellular immune responses in broilers challenged with *Clostridium perfringens* type A (1.9 × 10⁹ CFU/mL). As shown in Table 4, prophylactic and therapeutic groups exhibited significantly higher serum immunoglobulins (IgA, IgM), cytokines (IL-4, TNF-α), and T-cell markers (CD4⁺, CD8⁺) compared with the positive control (*p* ≤ 0.05). The most pronounced enhancement was observed in the prophylactic group, confirming that early administration of *E. faecium* strengthens immunity.

For example, IgM increased from 1.0 ± 0.20 mg/mL in the negative control to 10 ± 2.32 mg/mL in the prophylactic group, and CD8⁺ T cells increased from 180.5 ± 1.0 in the negative control to 450.7 ± 1.8 in the prophylactic group.

**Table 4:** Effect of *Enterococcus faecium* M74 on immunological parameters in broilers after *C. perfringens* type A infection (19th day).

Parameter	Negative Control	Positive Control	Prophylactic	Therapeutic
IgM (mg/mL)	1.0 ± 0.20 ^d^	2.8 ± 0.01 ^c^	10 ± 2.32 ^a^	9.1 ± 1.28 ^a^
IgA (mg/mL)	1.02 ± 0.12 ^d^	3.6 ± 0.02 ^c^	13 ± 0.13 ^a^	11.1 ± 0.16 ^b^
TNF-α (mg/dL)	2.52 ± 0.3 ^c^	5.61 ± 0.4 ^a^	4.88 ± 0.1 ^ab^	4.38 ± 0.16 ^b^
IL-4 (pg/mL)	220.5 ± 1.0 ^b^	230.3 ± 1.8 ^a^	230.7 ± 1.8 ^a^	228.4 ± 1.72 ^ab^
CD4⁺	600.5 ± 1.0 ^c^	1195.3 ± 1 ^a^	980.7 ± 1.8 ^ab^	960.4 ± 1.7 ^ab^
CD8⁺	180.5 ± 1.0 c	900.3 ± 1.8 a	450.7 ± 1.8 b	420.4 ± 1.7 b

Values are mean ± SE (n = 5). Different superscripts (a–d) indicate significant differences among groups (p ≤ 0.05, one-way ANOVA followed by Duncan’s multiple range test).

### Growth performance

Supplementation with *Enterococcus faecium* M74 (15 × 10⁹ CFU/kg) significantly improved growth performance in broilers infected with *Clostridium perfringens* type A (1.9 × 10⁹ CFU/mL). As shown in Table 5, the prophylactic group exhibited the highest final body weight (FBWT), body weight gain (BWG), and improved feed conversion ratio (FCR), followed by the therapeutic group. In contrast, the positive control group showed the poorest growth performance.

For example, FBWT increased from 1,465 ± 45.67 g in the positive control to 1,904 ± 39.79 g in the prophylactic group, while BWG improved from 1,430 ± 45.67 g to 1,880 ± 39.79 g. FCR decreased from 1.74 in the positive control to 1.30 in the prophylactic group, indicating more efficient nutrient utilization. These results suggest that *E. faecium* enhances growth and intestinal efficiency in infected broilers.

**Table 5:** Effect of *Enterococcus faecium* M74 on growth performance parameters in broilers after *C. perfringens* type A infection (19th day).

Parameter	Negative Control	Positive Control	Prophylactic	Therapeutic
Final body weight [FBWT] (g)	1,676 ± 47.66 ^b^	1,465 ± 45.67 ^c^	1,904 ± 39.79^a^	1,764 ± 34.34 ^a^
Body weight gain [BWG] (g)	1,631 ± 47.66 ^b^	1,430 ± 45.67 ^c^	1,880 ± 39.79^a^	1,720 ± 39.79 ^a^
Average feed intake [AFI] (g)	2,475 ± 5.01 ^ab^	2,495 ± 6.43 ^a^	2,310 ± 5.24 ^b^	2,260 ± 5.24 ^b^
Feed conversion ratio [FCR]	1.51 ^b^	1.74 ^a^	1.30 ^c^	1.31 ^c^

Values are mean ± SE (n = 5). Different superscripts (a–c) indicate significant differences among groups (p ≤ 0.05, one-way ANOVA followed by Duncan’s multiple range test).

### Intestinal morphometry (villus height and crypt depth)

Supplementation with *Enterococcus faecium* M74 (15 × 10⁹ CFU/kg) markedly improved intestinal morphology in broilers challenged with *Clostridium perfringens* type A (1.9 × 10⁹ CFU/mL). As shown in Table 6, both prophylactic and therapeutic groups exhibited significantly higher villus height and villus height/crypt depth ratio (*p* ≤ 0.05) compared with the positive control. The prophylactic group showed the most pronounced villus elongation, indicating enhanced absorptive capacity and improved intestinal health.

For example, villus height increased from 580.67 ± 64.46 μm in the positive control to 828.33 ± 56.37 μm in the prophylactic group, while the villus height/crypt depth ratio improved from 5.83 to 7.73, reflecting a more efficient intestinal surface for nutrient absorption.

**Table 6:** Effect of *Enterococcus faecium* M74 on villus height and crypt depth in broilers after *C. perfringens* type A infection (19th day).

Group	Villus Height (µm)	Crypt Depth (µm)	Villus Height/Crypt Depth Ratio
Negative Control	651.67 ± 56.51 ^b^	97.93 ± 16.47 ^c^	6.65 ^b^
Positive Control	580.67 ± 64.46 ^b^	89.65 ± 7.94 ^a^	5.83 ^c^
Prophylactic	828.33 ± 56.37 ^a^	107.97 ± 24.34 ^b^	7.73 ^a^
Therapeutic	676.18 ± 68.73 ^b^	92.81 ± 5.87 ^c^	7.28 ^a^

Values are mean ± SE (*n* = 5). Different superscripts (a–c) indicate significant differences among groups (*p* ≤ 0.05, one-way ANOVA followed by Duncan’s multiple range test).

### Histopathological lesion scoring

Microscopic examination of jejunal tissues revealed marked histopathological alterations in broilers challenged with *Clostridium perfringens* type A (1.9 × 10⁹ CFU/mL). The positive control group exhibited severe mucosal necrosis, villus erosion, and inflammatory cell infiltration. In contrast, broilers supplemented with *Enterococcus faecium* M74 (15 × 10⁹ CFU/kg) showed well-preserved intestinal architecture and reduced inflammation. The prophylactic group demonstrated near-normal morphology, with only minor degeneration, confirming the histoprotective effect of the probiotic.

**Fig. 1:**
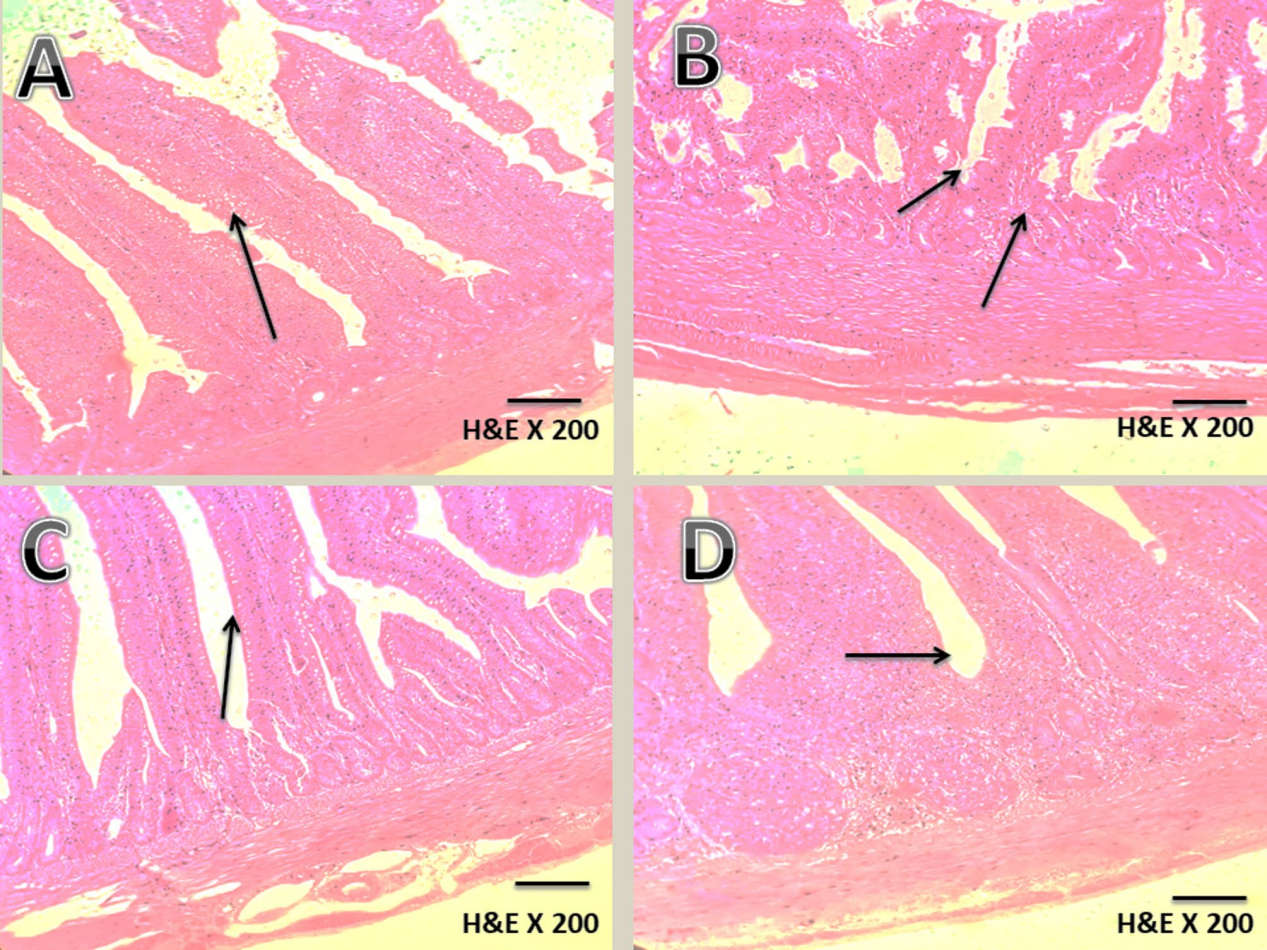
Representative jejunal micrographs (H&E ×200) after *C. perfringens* challenge.

As summarized in Table 7, congestion scores decreased from 3.2 ± 0.2 in the positive control to 0.8 ± 0.13 in the prophylactic group, while degeneration scores dropped from 3.4 ± 0.22 to 0.6 ± 0.22, reflecting substantial mucosal recovery. Representative micrographs of jejunal tissues are shown in Fig. 1, providing visual confirmation of these histological improvements.

**Table 7:** Effect of *Enterococcus faecium* M74 on histopathological lesion scoring of intestines in broilers after *C. perfringens* type A infection (19th day).

Parameter	Negative Control	Positive Control	Prophylactic	Therapeutic
Congestion	0 ± 0 ^c^	3.2 ± 0.2 ^a^	0.8 ± 0.13 ^b^	0.9 ± 0.26 ^b^
Degeneration	0.2 ± 0.13 ^c^	3.4 ± 0.22 ^a^	0.6 ± 0.22 ^b^	0.7 ± 0.18 ^b^

Values are mean ± SE (*n* = 5). Different superscripts (a–c) indicate significant differences among groups (*p* ≤ 0.05, one-way ANOVA followed by Duncan’s multiple range test).

- **Negative control**: Intact villi, continuous epithelial lining, normal mucosal architecture.
- **Positive control**: Villus necrosis, epithelial desquamation, and inflammatory infiltration in lamina propria, demonstrating severe mucosal damage.
- **Prophylactic** *E. faecium*: Broad villus tips, regenerated epithelial cells, intact lamina propria, reflecting marked structural improvement and mucosal protection.
- **Therapeutic** *E. faecium*: Numerous goblet cells, normalized villus height, minimal necrosis, indicating near-complete mucosal recovery.

### Bacterial re-isolation and intestinal microflora

Supplementation with *Enterococcus faecium* M74 (15 × 10⁹ CFU/kg) significantly reduced intestinal pathogen load in broilers challenged with *Clostridium perfringens* type A (1.9 × 10⁹ CFU/mL). As summarized in Table 8, both prophylactic and therapeutic groups exhibited significantly lower *C. perfringens* and total coliform counts (*p* ≤ 0.05) compared with the positive control.

This reduction in pathogenic bacteria was accompanied by a proportional increase in beneficial gut microflora, confirming the antimicrobial and microbiota-balancing effect of E. faecium. For example, total coliform counts decreased from 4.9 ± 0.1 CFU/mL in the positive control to 0.8 ± 0.19 CFU/mL in the prophylactic group, while intestinal bacterial counts dropped from 5.8 ± 0.1 to 0.7 ± 0.21 CFU/mL.

**Table 8:** Effect of *Enterococcus faecium* M74 on re-isolation of *C. perfringens* and intestinal bacterial counts in broilers (19th day).

Parameter	Negative Control	Positive Control	Prophylactic	Therapeutic
Total coliform counts (CFU/mL)	1.2 ± 0.1 ^b^	4.9 ± 0.1 ^a^	0.8 ± 0.19 ^c^	1.7 ± 0.23 ^b^
Intestinal bacterial count (CFU/mL)	3.2 ± 0.1 ^b^	5.8 ± 0.1 ^a^	0.7 ± 0.21 ^c^	2.3 ± 0.19 ^b^

Values are mean ± SE (*n* = 5). Different superscripts (a–e) indicate significant differences among groups (*p* ≤ 0.05, one-way ANOVA followed by Duncan’s multiple range test).

## Discussion

Poultry production represents the fastest-growing sector of the global livestock industry and is a major contributor to the world’s supply of animal protein. The industry’s remarkable expansion has been driven by genetic selection, improved nutrition, better management practices, and, historically, the use of antibiotics as growth promoters and prophylactic agents. However, the misuse and overreliance on antibiotics have led to antimicrobial resistance, creating a global health concern^16^. Hence, there is an urgent need to develop effective alternatives that maintain animal health and productivity while reducing antibiotic dependence.

Necrotic enteritis (NE), caused primarily by *Clostridium perfringens*, is one of the most economically devastating enteric diseases in poultry, leading to substantial global losses estimated at over two billion U.S. dollars annually^17,18^. *C. perfringens* is a ubiquitous, Gram-positive, spore-forming anaerobe found in soil, litter, water, and the gastrointestinal tracts of animals. The organism produces several toxins—particularly the necrotic enteritis beta toxin (NetB)—that destroy intestinal epithelial cells, disrupt gut homeostasis, and cause severe enteric damage^19–21^.

Predisposing factors such as coccidial infection, poor diet formulation, environmental stress, and intestinal mucosal damage further increase NE susceptibility. Clinically, birds show dehydration, depression, diarrhea, and characteristic necrotic lesions in the small intestine^22^. The prohibition of antibiotic growth promoters (AGPs) in many countries has increased the urgency of finding alternative strategies to control NE^23,24^.

### Probiotics as alternatives to antibiotics

Probiotics are live microorganisms that, when administered in adequate amounts, confer health benefits on the host^6^. They maintain gut microbial balance, improve nutrient utilization, and stimulate immune responses. *Enterococcus faecium*, a lactic acid bacterium commonly found in the intestinal tract, has emerged as one of the most effective probiotic candidates. The M74 strain has been approved as a feed additive by both the European Union and the U.S. FDA for its proven safety and beneficial effects on animal health^7,12^.

### Hematological findings

In the current study, infection with *C. perfringens* caused a significant decline in RBCs, Hb, and PCV, along with leukocytosis in the positive control group, indicating anemia and systemic inflammation. This aligns with^28^, who reported similar hematological alterations in infected broilers. Supplementation with *E. faecium* (M74), particularly in the prophylactic group, significantly restored hematological parameters toward normal values, suggesting enhanced oxygen transport capacity and improved immune competence.

### Liver and kidney function

Liver enzymes (AST, ALT, ALP) serve as key indicators of hepatocellular integrity. Elevated enzyme levels observed in the positive control group reflect hepatocyte damage and oxidative stress due to *C. perfringens* toxins. Birds receiving *E. faecium* showed marked reductions in enzyme activity and restored serum protein, albumin, and globulin levels, indicating hepatic protection and improved metabolic performance^29,30^. These findings are consistent with previous pharmacological studies on residue depletion and hemato-biochemical alterations in broilers^51^.

Similarly, elevated levels of urea, creatinine, uric acid, and potassium in infected birds demonstrated renal impairment. *E. faecium* supplementation normalized these parameters, suggesting nephroprotective activity, as previously noted by^31,32^. These effects may result from the probiotic’s ability to detoxify bacterial metabolites and maintain electrolyte balance.

### Immunological response

Immune parameters revealed a significant enhancement in IgA and IgM levels and elevated cytokines (IL-4, TNF-α) in the *E. faecium*–treated groups. The increased CD4⁺ and CD8⁺ T-cell counts indicate improved activation of both humoral and cellular immunity. These findings agree with^33–35^, who reported that *E. faecium* modulates cytokine secretion, enhances intestinal IgA production, and boosts mucosal immune defense.

Probiotic-induced immune modulation likely involves the stimulation of pattern recognition receptors, such as toll-like receptors (TLRs), leading to controlled activation of pro- and anti-inflammatory pathways. As^37,38^ reported, probiotics regulate tight junction genes and mucosal integrity, reducing intestinal permeability and pathogen invasion.

### Growth performance and feed efficiency

Birds supplemented with *E. faecium* exhibited significant improvements in body weight gain, feed intake, and feed conversion ratio (FCR). The prophylactic group showed the best performance, suggesting early administration provides maximal benefit. These findings align with those of^44–48^, who reported improved growth performance and nutrient absorption efficiency with probiotic supplementation. The improved feed conversion is likely due to enhanced digestion, better gut morphology, and reduced intestinal inflammation. Similar protective and growth-promoting effects have been reported with dietary supplementation using functional feed additives in aquatic species^52^.”

### Intestinal morphology and villus architecture

Histological analysis revealed substantial enhancement in intestinal villus height and villus height-to-crypt depth ratio in the *E. faecium* groups. These morphological improvements translate into larger absorptive surface areas, better nutrient assimilation, and improved mucosal integrity. The results are in line with^44,50^, who found that *E. faecium* supplementation increased villus height and decreased crypt depth in the jejunum and ileum.

### Histopathological and lesion scoring findings

Histopathological examination revealed that *C. perfringens* infection induced extensive villus necrosis, epithelial desquamation, and inflammatory infiltration. However, *E. faecium* supplementation, both prophylactic and therapeutic, significantly reduced lesion scores for congestion and degeneration. Previous pharmacological studies on lincomycin in broilers with necrotic enteritis support the observed histopathological improvements upon intervention^53^.

Along with increased villus length, only mild degenerative changes were observed across all treated groups. Examination of the cecum showed increased lymphoid elements post-infection, reflecting enhanced mucosal immune activation. Lesion scoring was performed using a standardized blinded method (0–4 scale: 0 = normal, 4 = severe). Recent studies have highlighted the potential beneficial effects of probiotics in mitigating necrotic enteritis lesions in broilers^54^.

These observations correspond with^39^, who reported that dietary *E. faecium* improved gut microvilli structure, promoted mucosal development, and influenced genes responsible for digestion, absorption, and epithelial maturation. Both *C. perfringens* and total coliform counts were significantly reduced in the E. faecium–treated groups, confirming its antibacterial and microbiota-modulating properties.

### Mechanistic insights

The beneficial effects of *E. faecium* (M74) can be attributed to several complementary mechanisms:

- Competitive exclusion of *C. perfringens* and other pathogens.
- Production of lactic acid and bacteriocins that inhibit harmful bacteria.
- Enhancement of mucosal immunity through cytokine modulation.
- Improvement in intestinal morphology and nutrient absorption.

Together, these mechanisms establish a healthier gut microenvironment, improve systemic immunity, and boost growth performance.

## Conclusion

Supplementation with *Enterococcus faecium* (M74) showed beneficial effects on broiler chickens challenged with *Clostridium perfringens* type A. The probiotic improved growth performance, hematological indices, liver and kidney functions, immune response, and intestinal morphology. Histopathological examination indicated reduced mucosal damage and enhanced tissue recovery, while intestinal bacterial counts of pathogens were significantly lowered.

Although these results highlight the protective and supportive role of *E. faecium* (M74), it should be considered primarily as a natural adjunct or prophylactic measure rather than a complete replacement for antibiotics. Further studies with larger sample sizes, longer experimental durations, and field trials are recommended to fully establish its potential as an alternative strategy for controlling necrotic enteritis in poultry.

## Data Availability

All data generated or analyzed during this study are included in this article. Additional raw data are available from the corresponding author upon reasonable request.

## Abbreviations

ALP: Alkaline phosphatase
ALT: Alanine aminotransferase
AST: Aspartate aminotransferase
BWG: Body weight gain
CAT: Catalase
COX-2: Cyclooxygenase-2
CRP: C-reactive protein
CFU: Colony-forming unit
*E. faecium*: *Enterococcus faecium*
FCR: Feed conversion ratio
GPx: Glutathione peroxidase
HIS: Hepatosomatic index
IgM: Immunoglobulin M
IL-1β: Interleukin-1 beta
IL-10: Interleukin-10
iNOS: Inducible nitric oxide synthase
NF-κB: Nuclear factor kappa-light-chain-enhancer of activated B cells
NO: Nitric oxide
NE: Necrotic enteritis
PER: Protein efficiency ratio
RBCs: Red blood cells
ROS: Reactive oxygen species
SOD: Superoxide dismutase
TGF-β1: Transforming growth factor beta-1
TLC: Total leukocytic count
TNF-α: Tumor necrosis factor-alpha
WBCs: White blood cells
